# Machine learning classification of polycystic ovary syndrome based on radial pulse wave analysis

**DOI:** 10.1186/s12906-023-04249-5

**Published:** 2023-11-13

**Authors:** Jiekee Lim, Jieyun Li, Xiao Feng, Lu Feng, Yumo Xia, Xinang Xiao, Yiqin Wang, Zhaoxia Xu

**Affiliations:** 1 School of Traditional Chinese Medicine, Shanghai University of Traditional Chinese Medicine, Shanghai, 201203 P. R. China; 2 The First Affiliated Hospital, Guangzhou University of Traditional Chinese Medicine, Guangzhou, 510405 P. R. China; 3 Shanghai Key Laboratory of Health Identification and Assessment, Shanghai, 201203 P. R. China

**Keywords:** Machine learning, Polycystic ovary syndrome, Pulse diagnostics, Radial pulse wave, Traditional Chinese medicine

## Abstract

**Background:**

Patients with Polycystic ovary syndrome (PCOS) experienced endocrine disorders that may present vascular function changes. This study aimed to classify and predict PCOS by radial pulse wave parameters using machine learning (ML) methods and to provide evidence for objectifying pulse diagnosis in traditional Chinese medicine (TCM).

**Methods:**

A case-control study with 459 subjects divided into a PCOS group and a healthy (non-PCOS) group. The pulse wave parameters were measured and analyzed between the two groups. Seven supervised ML classification models were applied, including K-Nearest Neighbors (KNN), Support Vector Machine (SVM), Decision Trees, Random Forest, Logistic Regression, Voting, and Long Short Term Memory networks (LSTM). Parameters that were significantly different were selected as input features and stratified k-fold cross-validations training was applied to the models.

**Results:**

There were 316 subjects in the PCOS group and 143 subjects in the healthy group. Compared to the healthy group, the pulse wave parameters h3/h1 and w/t from both left and right sides were increased while h4, t4, t, As, h4/h1 from both sides and right t1 were decreased in the PCOS group (*P* < 0.01). Among the ML models evaluated, both the Voting and LSTM with ensemble learning capabilities, demonstrated competitive performance. These models achieved the highest results across all evaluation metrics. Specifically, they both attained a testing accuracy of 72.174% and an F1 score of 0.818, their respective AUC values were 0.715 for the Voting and 0.722 for the LSTM.

**Conclusion:**

Radial pulse wave signal could identify most PCOS patients accurately (with a good F1 score) and is valuable for early detection and monitoring of PCOS with acceptable overall accuracy. This technique can stimulate the development of individualized PCOS risk assessment using mobile detection technology, furthermore, gives physicians an intuitive understanding of the objective pulse diagnosis of TCM.

**Trial registration:**

Not applicable.

**Supplementary Information:**

The online version contains supplementary material available at 10.1186/s12906-023-04249-5.

## Background

Polycystic ovary syndrome (PCOS) is one of the most common gynaecological endocrine disorders in women of reproductive age, affecting 5–20% of women worldwide [1]. PCOS is characterized by ovulatory dysfunction, hyperandrogenism, and polycystic ovarian morphology (PCOM). PCOS increases the risk of metabolic complications, cardiovascular disease, endometrial cancer, and mental health disorders [2–4]. The economic burden of PCOS is estimated at USD 8 billion annually [5]. Therefore, early diagnosis of PCOS is essential to prevent the long-term complications of the disease and to reduce the medical burden.

**Fig. 1:**
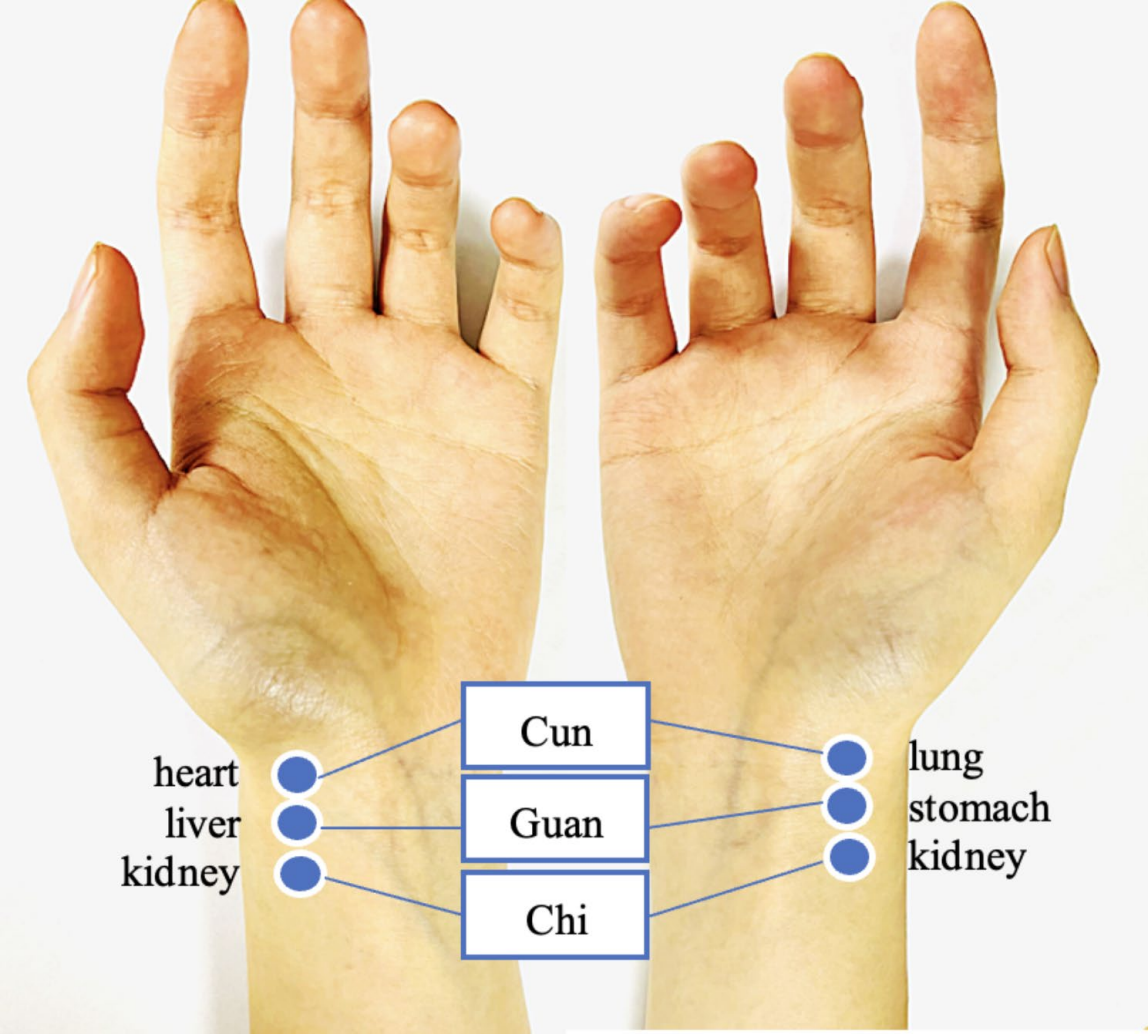
Pulse diagnosis on the radial artery and the corresponding internal organ systems. Pulse diagnosis is done by palpating three adjacent regions of the wrist of both hands, namely Cun (distal), Guan (middle), and Chi (proximal) position

Pulse diagnosis, which is a non-invasive, convenient, and simple method, is one of the most common diagnostic methods in TCM. It is done by palpating the radial artery pulse as shown in Fig. 1, each pulse position is a reflection point of a certain internal organ system [6]. For centuries, the practitioner gains insights into patients’ physical conditions and constitutions by interpreting the characteristics of the pulse, the pulse can reflect the conditions of internal organs, Qi (vital energy), and blood of individuals [7].

However, traditional pulse diagnosis relies on the palpation sensitivity of the practitioners, the judgments are always limited due to subjective observations based on one’s experience. Consequently, the importance of objectifying and quantifying radial pulse waves has been drawing attention [8, 9]. Previous studies suggested that, compared to healthy individuals, changes in radial pulse wave in PCOS are related to hemodynamic changes, ventricular systolic function, and aortic compliance in the cardiovascular system [10, 11], and the pulse wave parameters varied at different body mass index (BMI) levels in PCOS [12].

Merely understanding specific relevant factors is not enough in actual clinical diagnosis and treatment of PCOS, to better serve the clinical needs, the application of ML algorithms for diagnosis and predictions has been reported using different features, such as Raman spectroscopy of follicular fluid [13], PCOS genes [14, 15], ovary ultrasound images and reports [16, 17], or clinical data set [18–20]. However, the study about the ML prediction model of PCOS based on pulse wave parameters has not been found yet. Some evidence supported that ML models using pulse waves are of great significance in disease predictions [21–25]. Therefore, through the comparisons of ML prediction models, we intended to determine whether PCOS could be diagnosed and monitored by radial pulse wave.

The objective of this study is to classify and predict PCOS by radial pulse wave parameters using machine learning methods and to provide evidence for objectifying pulse diagnosis in TCM.

## Methods

The study followed the Transparent Reporting of a Multivariable Prediction Model for Individual Prognosis or Diagnosis (TRIPOD) guidelines.

### Study subjects

This case-control study involved two groups. The PCOS group included 316 patients with PCOS while the healthy group included 143 normal subjects. All the participants were recruited from the Shanghai University of TCM (SHUTCM), Shanghai Municipal Hospital of Traditional Chinese Medicine, Shuguang Hospital, and Yueyang Hospital affiliated with SHUTCM, from August 2018 to January 2022.

#### Inclusion criteria

Female participants aged 18–40 years old were eligible for this study. Based on “Chinese guidelines for diagnosis and treatment of polycystic ovary syndrome (2018)” [26], the diagnosis of PCOS was as follows:

- oligomenorrhea or amenorrhea or irregular uterine bleeding is a necessary condition.
- 1 of the following 2 criteria must be met: clinical and/or biochemical hyperandrogenism (HA), polycystic ovarian morphology (PCOM).

Participants in the healthy group were required to show none of the PCOS criteria and were free from gynaecological and organic diseases. All participants must sign the written informed consent.

#### Exclusion criteria

The exclusion criteria were as follows:

- other diseases that may cause hyperandrogenism and abnormal ovulation;
- participants with other apparent gynaecological diseases and organic diseases such as liver or kidney disorders;
- participants with adenomyosis, Cushing’s syndrome, chromosomal abnormalities, congenital adrenal cortical hyperplasia, and chocolate cyst of the ovary;
- participants with serious primary diseases in internal medicine and surgery;
- patients with significant incomplete clinical data;
- patients who were unable to cooperate.

### Radial pulse signal collection

The pulse signals were collected from the Guan position of participants’ left and right hands using the Z-BOX pulse meter, radial pulse signals can be detected most clearly and easily at the Guan position. The time of collection was from 9 AM to 11 Am or from 1 PM to 4:30 PM. The participants were required to keep calm and prohibited to eat and drink 30 min before the test, they also had to avoid violent mood swings. During the test, the participant was required to breathe calmly, sit upright, keep the left arm relaxed, and spread forward the left forearm naturally, the wrist was placed on a pulse pillow with the palm facing up and the fingers slightly bent. The Z-BOX pulse meter was attached to the wrist where the pressure sensor was placed on Guan position, in the meantime, the participant should avoid speaking or moving. A series of radial pulse signals within the pulse pressure range of 25–250 g were recorded continuously for 30s. The sampling process repeats with the right arm. The radial pulse signal with the highest main amplitude, apparent fluctuation of three peaks, and a steep ascending branch without incisure was selected for time-domain parameter analysis. As pulse signals can be affected by a variety of noise sources, including patient tremors, respiration, mechanical vibrations of instruments, and power frequency interference. We used the PulseSystem software [jointly developed by our research group and East China University of Science and Technology (Shanghai)] to de-noise pulse signals and extract the pulse wave parameters. Pulse signals are concentrated in the low-frequency range, so the software used a Butterworth filter to remove high- and low-frequency noise. The filter order was set to 3, and the passband range was set to 0.2–20 Hz. To prevent bias, the pulse meter used for data collection was consistent, and data collection was done by the same executors (XF and LF) with adequate training, double entry and verification are adopted for data entry by the same executors again.

### Time-domain parameters of the radial pulse wave signal

**Fig. 2:**
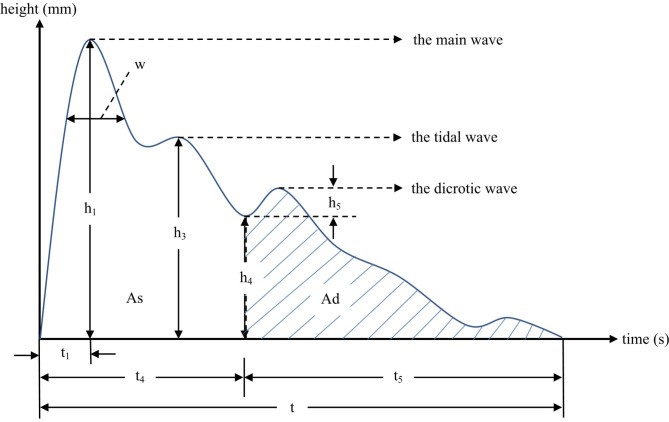
The amplitude, time, and area parameters of the radial pulse diagram

**Fig. 3:**
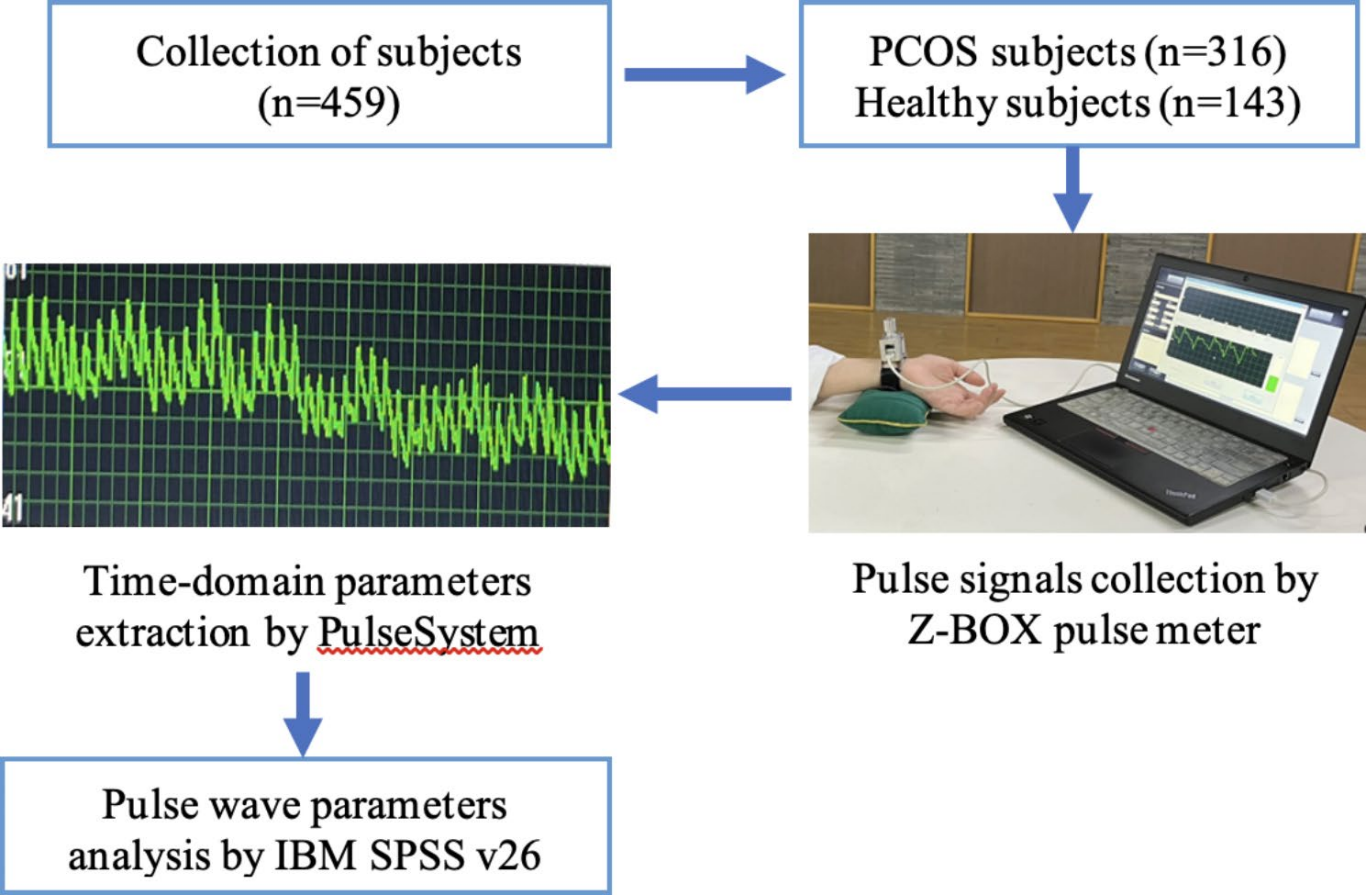
Flowchart of pulse wave parameters collection and analysis

Figure 2 shows the time-domain parameters which are commonly used in TCM radial pulse wave analysis. 30 time-domain parameters were extracted for comparisons, including h1, h3, h4, h5, t1, t4, t5, t, w, As, Ad, h3/h1, h4/h1, h5/h1, w/t, from both left and right Guan position. The interpretation of the meaning of pulse wave parameters was done by referring to “Pulse Diagnosis of Modern TCM” [27], each parameter corresponded to specify physiological significance. Parameters h1, h3, h4, and h5 are the main wave, tidal wave, dicrotic notch, and dicrotic wave amplitude accordingly. Parameter t is the time for a complete pulse cycle, t1 is the time between the starting point to the crest of the main wave, t4 is the time between the starting point to the dicrotic notch, t5 is the time between the dicrotic notch to the ending point. Parameter w is the width of the main wave at its 1/3 height. As is the area of the systolic phase while Ad is the area of the diastolic phase. The ratio h3/h1 reflects vascular wall compliance and peripheral resistance, h4/h1 reflects the level of peripheral resistance, h5/h1 mainly reflects aortic compliance and aortic valve function, and w/t corresponds to the duration of elevated aortic pressure and is related to peripheral resistance. Figure 3 showed the flowchart of pulse wave parameters collection and analysis.

### Statistical analysis of pulse wave parameters

Statistical analysis of radial pulse wave parameters was performed using IBM SPSS Statistics (version 26). All measurements from the two groups (PCOS patient group and healthy control group) did not conform to normal distribution. Therefore, independent samples Wilcoxon rank sum test (Mann-Whitney U test) was used to compare between groups. The results were presented by median, M(P_25_, P_75_). The level of statistical significance was set at *P* < 0.05 for all the analyses.

### Machine learning classification method

**Fig. 4:**
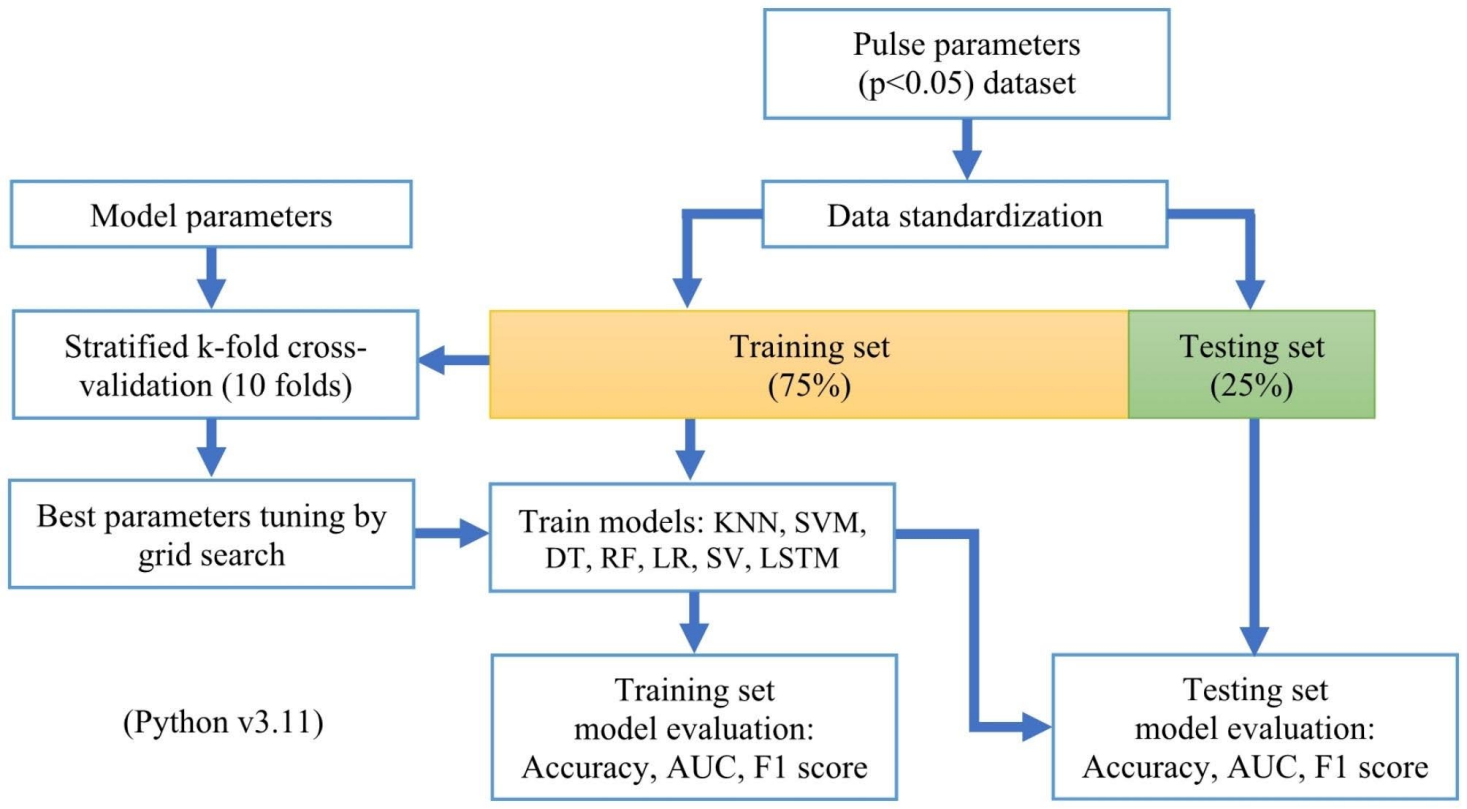
Flowchart of PCOS and healthy group discrimination by machine learning classifiers. 15 parameters (*P* < 0.05) were used as the features for model training

Machine Learning (ML) classification is used to predict categories, which are the PCOS group and the healthy group here. Figure 4 shows the flowchart of the machine learning classification process.

Supervised learning is used for the study, it is a type of machine learning where the predicted values are already known. Given the features and target variables, a model is built to accurately predict target values of unseen data, in our case, predict a subject’s class based on pulse wave parameters (*P* < 0.05). Seven supervised machine learning classifiers were used to build models, including K-Nearest Neighbors (KNN), Support Vector Machine (SVM), Decision Trees (DT), Random Forest (RF), Logistic Regression (LR), Soft Voting (SV), and Long Short Term Memory networks (LSTM).

The pulse wave parameters dataset was split into a training set (75%) and a testing set (25%). The training set was used for model training and the testing set was used to evaluate final model performance. Data splitting is a crucial step to prevent overfitting since ML classifiers can perform relatively well on trained data [28]. Data standardization of features was done by subtracting the mean and dividing by variance so that all features were centred around zero and had a variance of one.

Stratified k-fold cross-validation was applied to the training data, the data were further split into train/test sets for 10 folds, the folds are made by preserving the percentage of subjects for each class, this technique is good for an imbalanced class as in our case. Models were trained using the seven classifiers, the best model parameters were tuned by the grid search. The model performance based on the training data was evaluated by accuracy, area under the ROC curve (AUC), and F1 score, then, the evaluation was repeated on the testing set. The final evaluation aimed to check the general ability of models to predict unseen data.

#### Machine learning classifiers

K-Nearest Neighbors (KNN) is a non-parametric algorithm that is popular for classification problems. KNN uses “k” numbers of closest labelled data points to predict new data points, prediction is made based on the majority labels of the nearest neighbours. The determined value of k is 22 in this study.

Support Vectors Machines (SVM) work by finding the hyperplane that maximally separates the data points of different classes [29]. We applied the SVM radial basis function (RBF) kernel which is suitable for nonlinear data in this study.

Decision Trees (DT), also known as Classification and Regression Trees (CART)), can be used to predict categorical or continuous outcomes, which are commonly used in epidemiological and medical fields [30]. When a classification tree is trained, the tree learns a sequence of if-else questions about individual features to infer the class labels. The maximum depth determined by grid search was 2 here in this study.

Random Forest (RF) is one of the best ensemble learning methods of decision trees. Random Forest uses random subsamples of training data and randomizing the algorithm for base-level classifiers (decision trees), a subset of features is randomly selected by decision trees and the best is chosen among these at each step of tree construction [31]. Random Forest models are less prone to overfitting and can achieve higher accuracy in disease prediction. A Random Forest with 300 decision trees and a maximum depth of 4 was determined in this study.

Logistic Regression (LR) calculates the probability of an observation belonging to the binary class. The predicted probability is compared to the default probability threshold to make the classification.

A voting classifier with soft voting (SV) is chosen, the model trains on the ensemble of the five models above (KNN, SVM, DT, RF, LR), and the class label is predicted based on the highest average probability given to that class. This ensemble-based Voting classifier is expected to improve model performance compared to a single classifier [32].

Long Short-Term Memory networks (LSTM) are a type of recurrent neural network (RNN) architecture specifically designed to capture and process sequential and time series data, which have been widely used in speech recognition, natural language processing, and time series prediction [33, 34]. While a simple RNN can use past predictions to infer new ones, LSTMs were introduced to overcome the limitations of RNN, which hard to manage long-range dependencies due to the vanishing gradient problem [35]. After parameters tuning, we built the LSTM model with 2 LSTM layers and 1 Dense layer, each followed by a Dropout layer, lastly an output layer. The model architecture was stated in the supplementary material 1.

#### Classification metrics

In the present study, the main metrics used for model performance evaluation are accuracy, F1 score, and AUC.

**Fig. 5:**
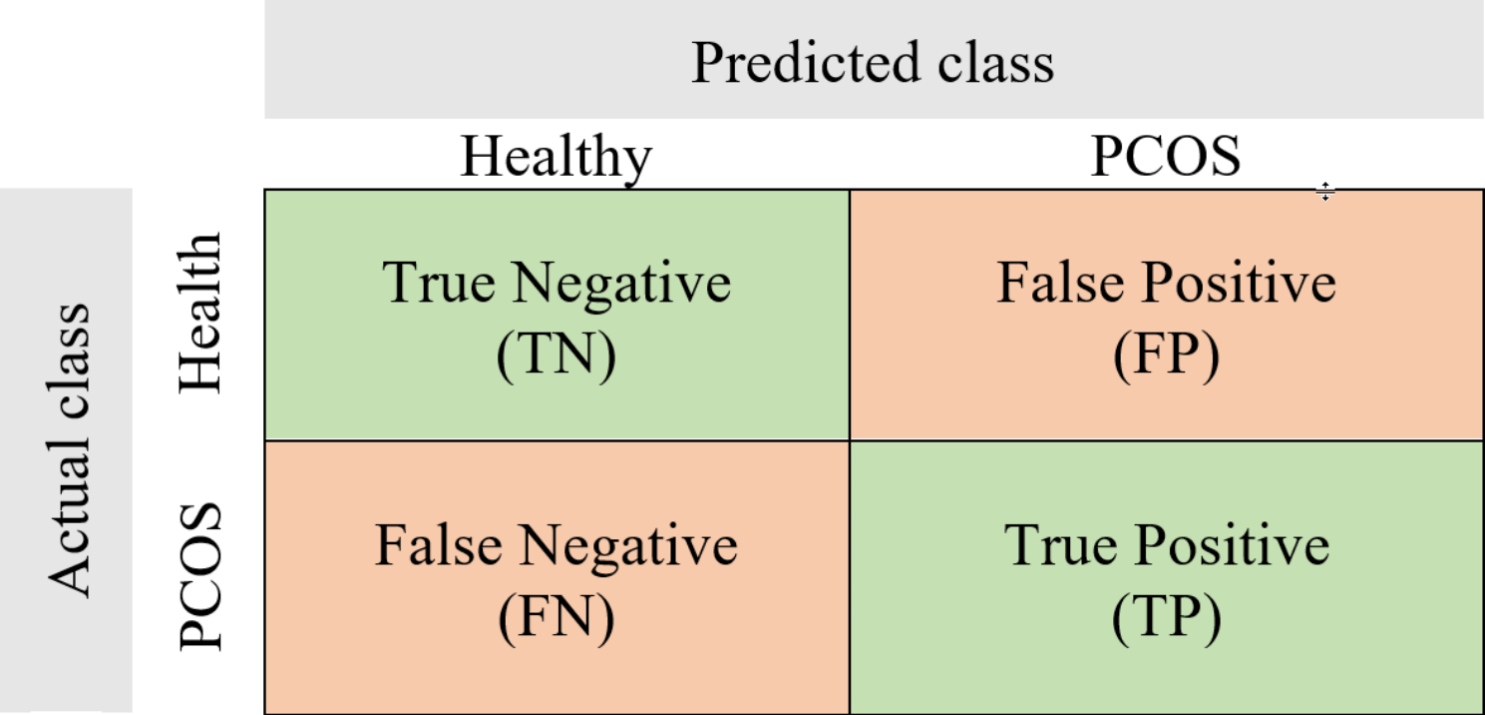
The confusion matrix

The confusion matrix can summarize the model performance as in Fig. 5. The true positives (TP) are the number of PCOS subjects correctly predicted; the true negatives (TN) are the number of healthy subjects correctly predicted; the false negatives (FN) are the number of healthy subjects incorrectly predicted; and the false positives are the number of PCOS subjects incorrectly predicted.

Accuracy is the proportion of the correct predictions to the total number of observations. It is a commonly used metric to measure model performance in classification.

$$Accuracy =\frac{TN+TP}{TN+FN+FP+TP}$$

Precision is the positive predictive value. It is the ratio of true positives to the sum of all positive predictions. High precision means having a lower false positive rate.

$$Precision =\frac{TP}{TP+FP}$$

Recall is the ratio of true positives to the sum of true positives and false negatives. It is also called sensitivity. High recall reflects a lower false negative rate. For the present study, high recall means predicted most PCOS subjects correctly.

$$Recall =\frac{TP}{TP+FN}$$

F1 score is the harmonic mean of precision and recall, thus, it evaluates a model’s precision and recall ability. F1 score ranges from 0 (worst) to 1 (best).

$$F1 score =2\times \frac{Precision\times Recall}{Precision+Recall}$$

Area under the Receiver Operating Characteristic curve (AUC/ ROC AUC), is a useful metric to visualize and evaluate classification ability [36]. ROC graph reveals the relationship between true positive rate (TPR) and false positive rate (FPR). AUC ranges from 0 to 1.0, 0.5 means random guessing, the larger the AUC the better the model is.

## Results

### Baseline characteristics

**Fig. 6:**
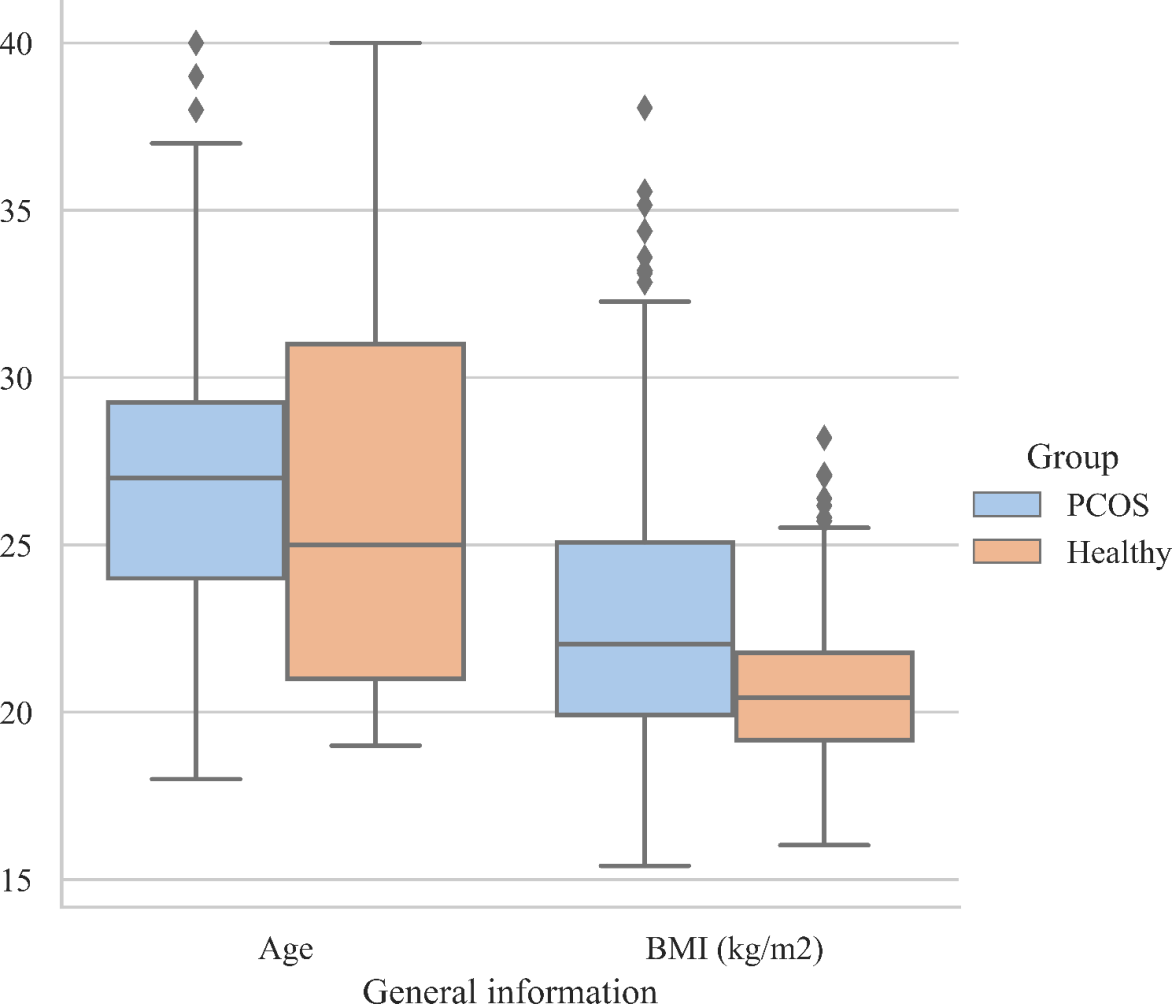
Box plot of age and BMI distribution

Table 1; Fig. 6 show the comparisons of age and BMI of the subjects from the PCOS group and healthy group. Subjects from different groups did not show significant differences in age (*P* > 0.05) but showed significant differences in BMI (*P* < 0.01). The BMI of the PCOS group was significantly higher than the healthy group.

**Table 1:** Comparison of general information (n = 459)

Group	Age	BMI (kg/m^2^)
PCOS (*n* = 316)	27.00 (24.00, 29.75)	22.03 (19.91, 25.08)
Healthy (*n* = 143)	25.00 (21.00, 31.00)	20.44 (19.15, 21.77)
*P*	0.061	< 0.01*

*P < 0.01

### Comparisons of time-domain parameters

The time-domain parameters of pulse diagrams from both left and right Guan positions were compared between the PCOS group and healthy control group using the Wilcoxon rank sum test. Tables 2 and 3 showed the results of the comparisons. For both the left and right sides, compared to the healthy group, the parameters h4, t4, t, As, h4/h1 were significantly lower in the PCOS group (*P* < 0.01) while the parameters h3/h1 and w/t were significantly higher in PCOS group (*P* < 0.01). The right t1 was significantly lower in the PCOS group compared to the healthy group (*P* < 0.01). No significant differences were observed in the rest of the pulse wave parameters between the groups (*P* > 0.05).

**Table 2:** Comparison of time-domain pulse wave parameters from left Guan position

Time domain parameters (left)	PCOS group (*n* = 316)	Healthy group (*n* = 143) (*n* = 143)	MannWhitney U Test	*P*
h1	51.1463(36.1461,62.1430)	51.0814(37.7732,68.6276)	20663.00	0.14
h3	42.0940(29.3900,51.8107)	39.9151(29.1578,52.5250)	22553.50	0.98
h4	12.2748(4.9953,20.7704)	17.0547(7.1223,26.6643)	18800.50	< 0.01**
h5	25.9800(16.5715,35.0660)	26.3911(16.6583,36.7083)	22180.00	0.75
t1	0.0436(0.0411,0.0478)	0.0439(0.0413,0.0473)	22432.00	0.9
t4	0.0665(0.0475,0.0849)	0.0810(0.0573,0.0960)	17108.50	< 0.01**
t5	0.1560(0.1320,0.1791)	0.1575(0.1326,0.1865)	21420.50	0.37
t	0.2199(0.2036,0.2416)	0.2351(0.2158,0.2572)	16446.00	< 0.01**
w	0.0616(0.0505,0.0706)	0.0577(0.0484,0.0688)	20269.50	0.08
As	1110.1580(562.0087,1820.6702)	1540.8545(726.5162,2432.4840)	18174.00	< 0.01**
Ad	2051.2545(1322.9600,3009.1210)	2042.1479(1306.9180,2853.1460)	22224.00	0.78
h3/h1	0.8620(0.7703,0.9068)	0.8056(0.7113,0.8768)	17230.00	< 0.01**
h4/h1	0.2716(0.1384,0.3915)	0.3533(0.1886,0.4463)	18570.00	< 0.01**
h/5h1	0.5197(0.4133,0.7125)	0.5021(0.3787,0.6911)	21547.00	0.43
w/t	0.2781(0.2431,0.3124)	0.2478(0.2119,0.2867)	15750.00	< 0.01**

*P < 0.05 **P < 0.01

**Table 3:** Comparison of time-domain pulse wave parameters from the right Guan position

Time domain parameters (right)	PCOS group (*n* = 316)	Healthy group (*n* = 143) (*n* = 143)	MannWhitney U Test	*P*
h1	53.6468(39.4475,64.9978)	56.3246(41.1000,71.4271)	20346.00	0.09
h3	41.0358(30.5258,51.8072)	40.3392(29.0920,53.4807)	22365.00	0.86
h4	14.8259(7.1823,23.1606)	20.3658(11.8300,29.3906)	17541.00	< 0.01**
h5	23.8942(14.4083,36.8861)	26.5248(17.3289,34.6542)	21196.00	0.29
t1	0.0426(0.0405,0.0458)	0.0441(0.0415,0.0472)	19140.50	< 0.01**
t4	0.0752(0.0570,0.0905)	0.0929(0.0730,0.1014)	15079.00	< 0.01**
t5	0.1469(0.1233,0.1702)	0.1480(0.1289,0.1708)	21916.50	0.61
t	0.2199(0.2036,0.2416)	0.2351(0.2158,0.2572)	16446.00	< 0.01**
w	0.0556(0.0459,0.0660)	0.0523(0.0416,0.0651)	20568.50	0.12
As	1413.7820(762.0707,2039.2582)	1892.5390(1175.0580,2725.4790)	16729.00	< 0.01**
Ad	1961.8885(1163.8118,2810.0853)	1729.3390(1201.8840,2604.1810)	22138.00	0.73
h3/h1	0.8117(0.7123,0.8798)	0.7249(0.6472,0.8534)	17282.00	< 0.01**
h4/h1	0.3224(0.1935,0.4141)	0.3866(0.2749,0.4459)	17573.00	< 0.01**
h5/h1	0.4689(0.3546,0.6285)	0.4862(0.3920,0.5685)	22082.00	0.7
w/t	0.2582(0.2212,0.2844)	0.2308(0.1792,0.2741)	16914.00	< 0.01**

*P < 0.05 **P < 0.01

### Machine learning classification based on radial pulse wave parameters

**Fig. 7:**
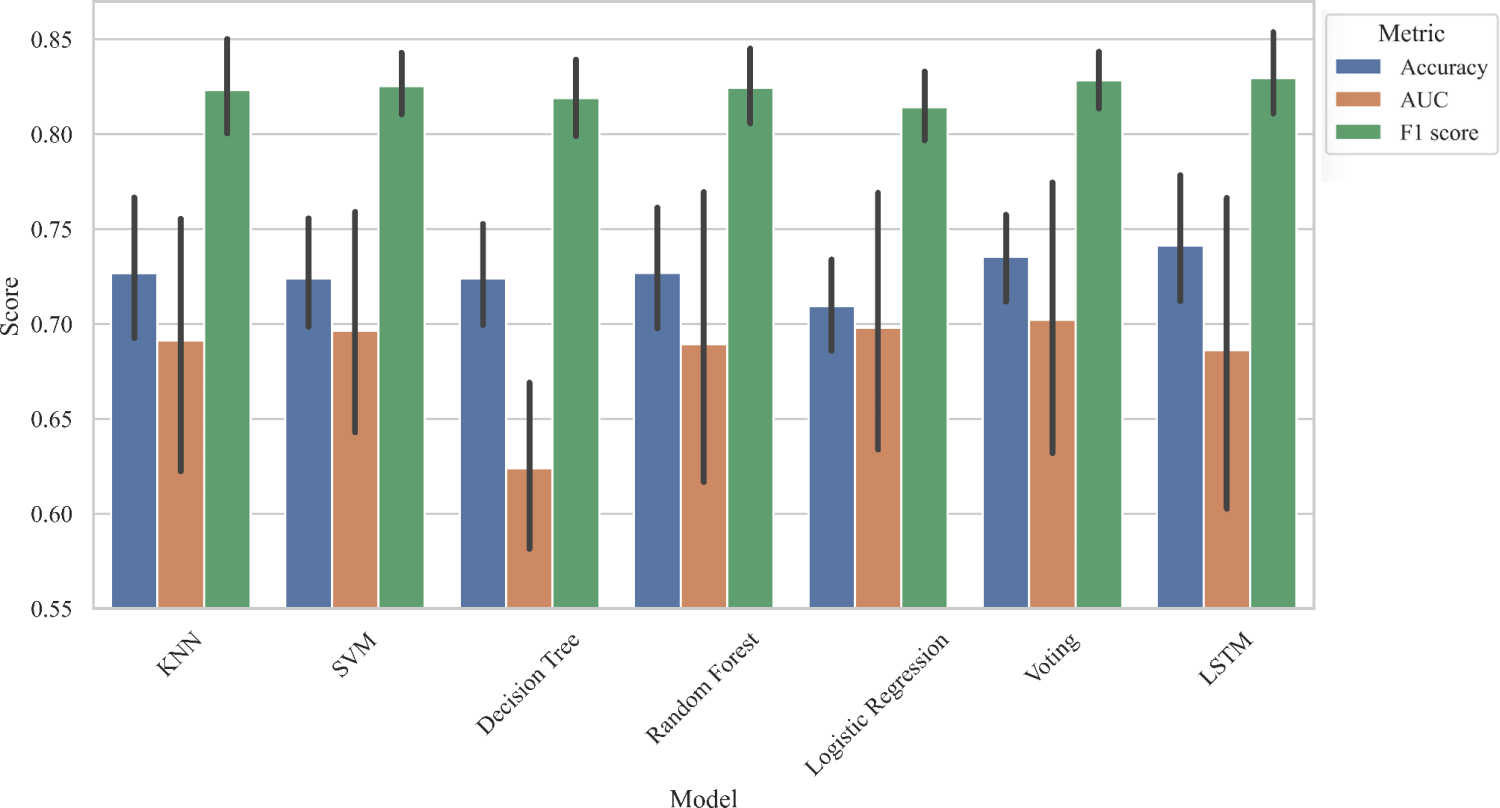
Stratified k-fold cross-validation training results of machine learning models

**Fig. 8:**
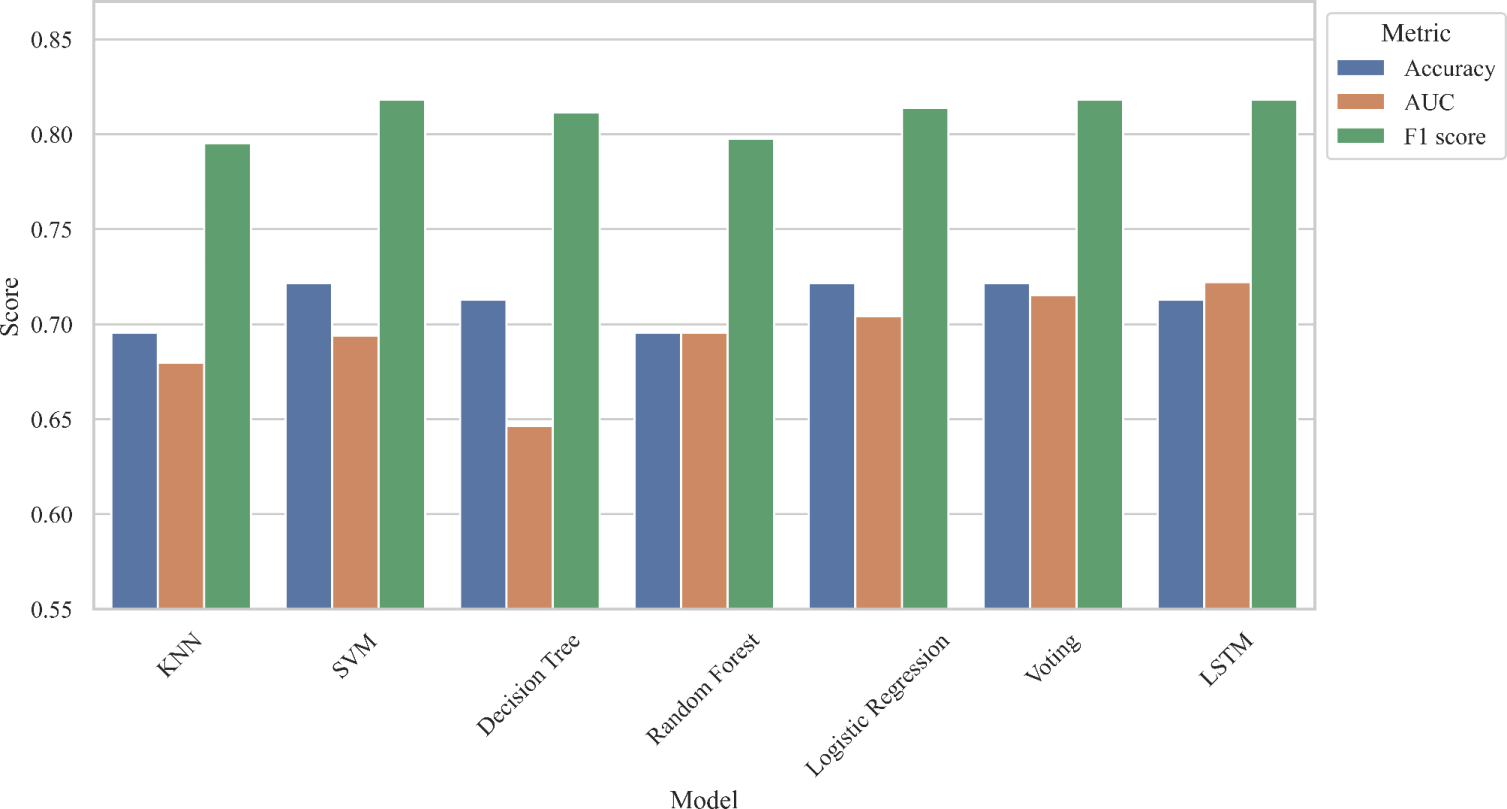
Testing results of machine learning models

15 parameters (*P* < 0.05) were selected as feature variables for model training including right t1 and h4, t4, t, As, h4/h1, h3/h1, w/t from both left and right sides. The target variables were the groups of the subjects. The performance metrics including accuracy, AUC, and F1 score were calculated, the summary of results is presented in Table 4, Fig. 7 and 8.

**Fig. 9:**
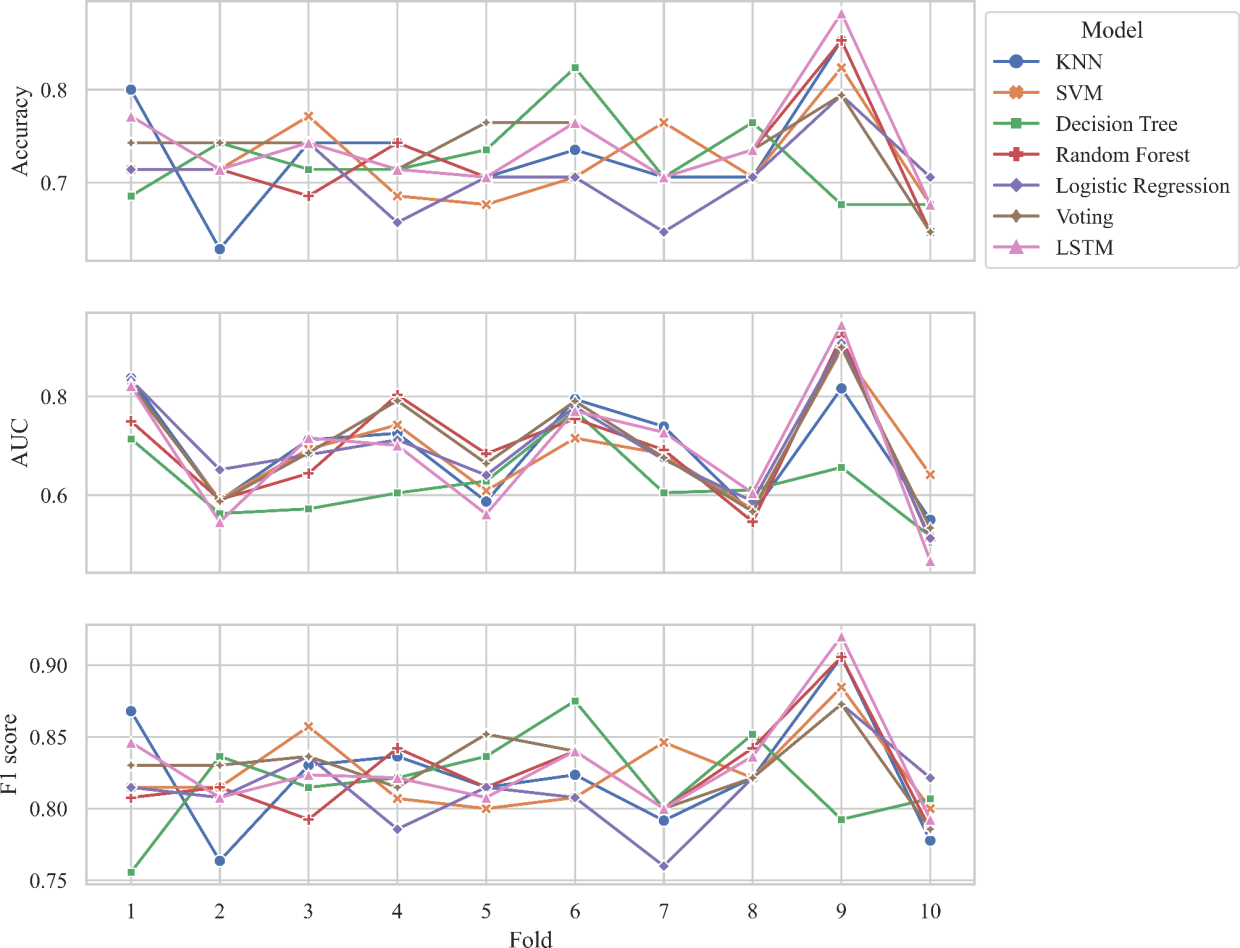
Results of stratified k-fold cross-validation for each fold, accuracy, AUC, and F1 score are visualized respectively

For the stratified k-fold cross-validation training, the result showed that KNN, SVM, Decision Trees, and Random Forest performed similarly by gaining similar accuracy scores. They have slightly lower performance compared to LSTM and Voting ensemble. LSTM achieved the highest accuracy and AUC at 74.135 ± 5.437 and 0.702 ± 0.115 respectively while Voting achieved the highest F1 score at 0.831 ± 0.027. The cross-validation results for each fold are visualized in Fig. 9.

**Fig. 10:**
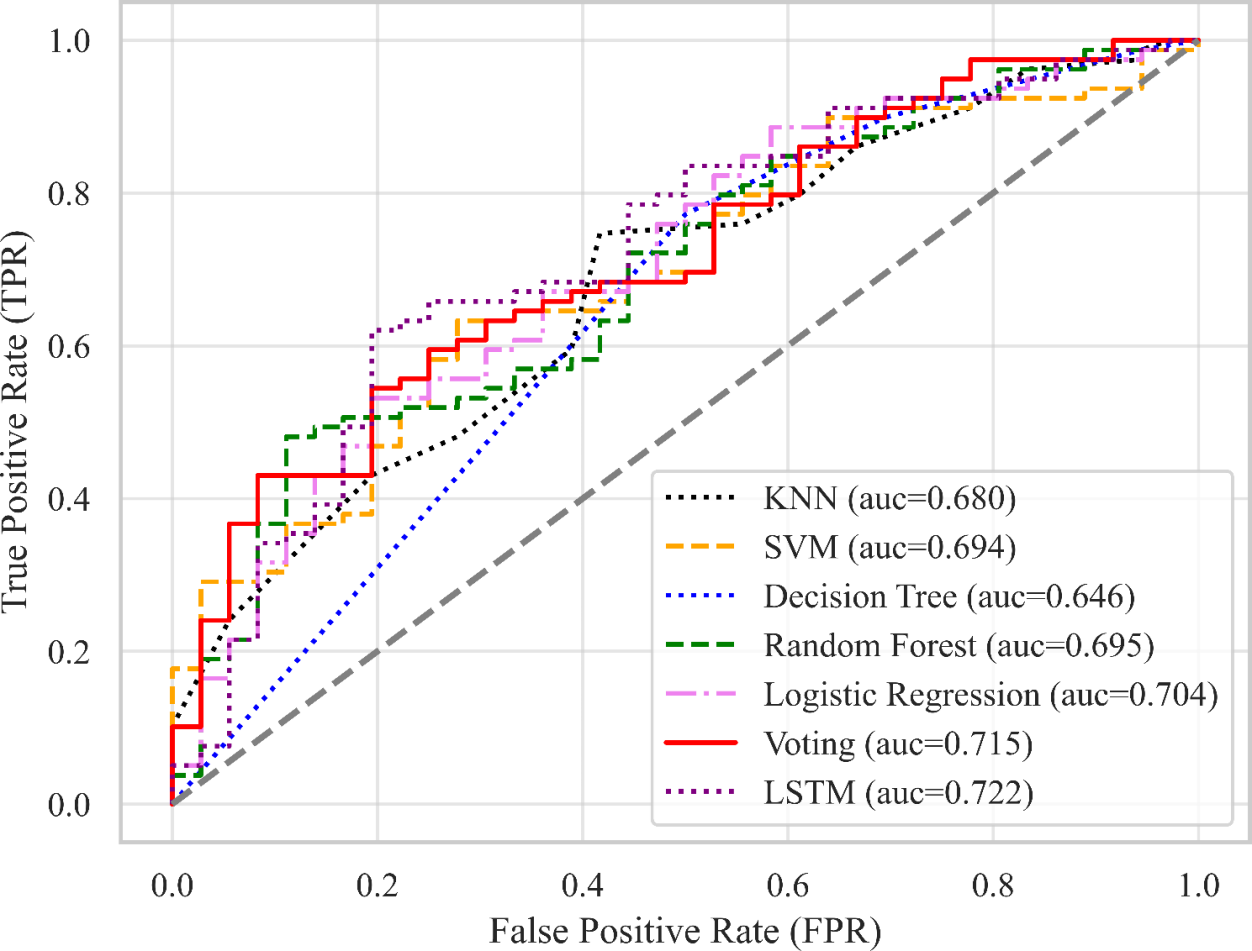
Testing scores of Area under the Receiver Operating Characteristic curve (AUC).

**Fig. 11:**
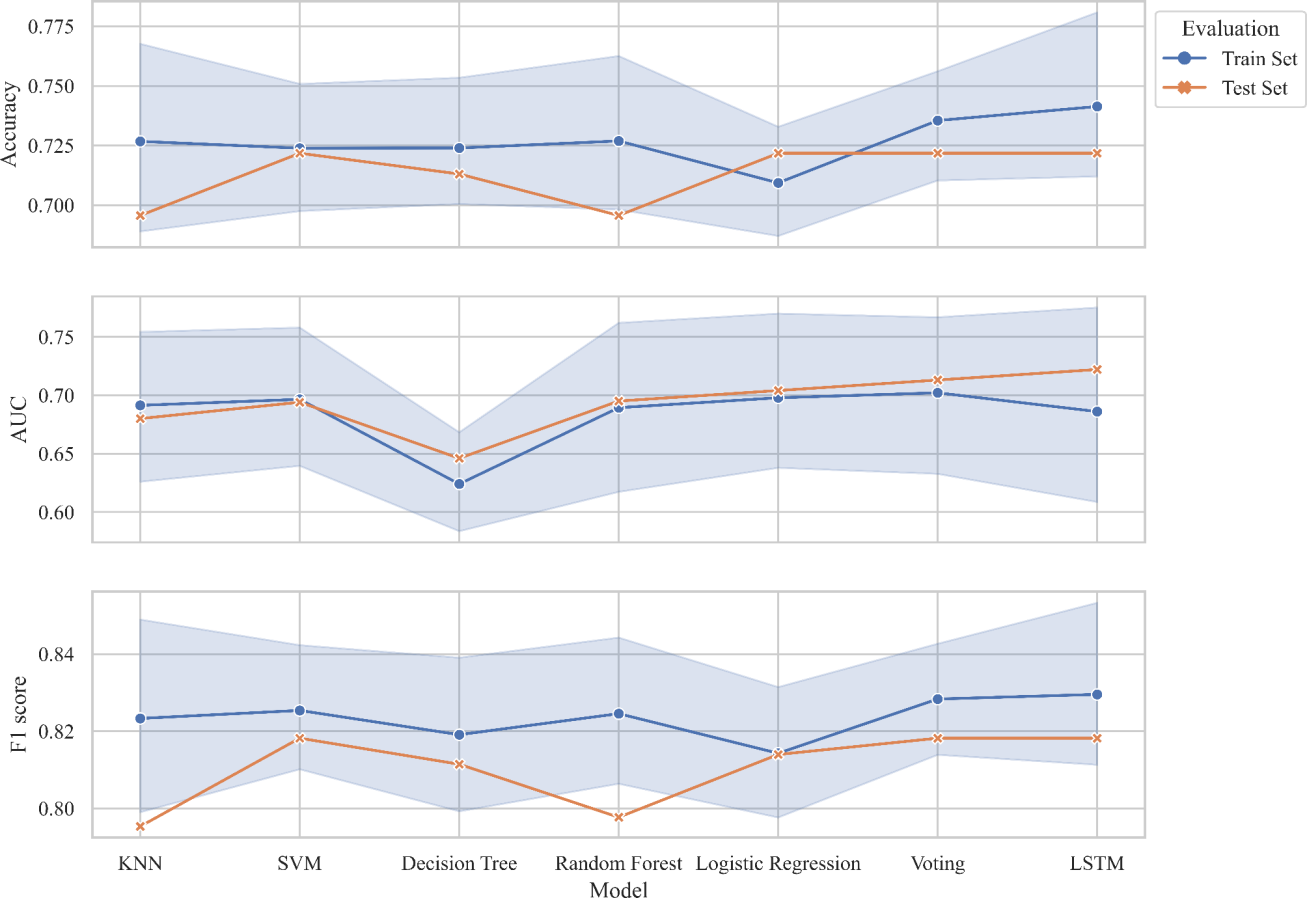
Model performance comparisons of training results and testing results. Training results came from the training data that was used for both training and testing while testing results came from the ability of the trained model to identify unseen testing data that were not used in training

For the testing set evaluation, SVM, Logistic Regression, Voting, and LSTM achieved the highest accuracy (72.17%). SVM, Voting, and LSTM got the highest F1 score (0.818), LSTM again performed the best for AUC (0.722), thus, LSTM had the best testing performance. Figure 10 showed the AUC of the ROC graph among models and Fig. 11 compared the performance metrics between the training set and testing set. Both the Voting and LSTM exhibited similar levels of performance across all metrics.

**Table 4:** Performance evaluation of models

Models	Training results (Stratified k-fold cross-validations)			Testing results		
	Accuracy	AUC	F1 score	Accuracy	AUC	F1 score
KNN	72.672 ± 6.259	0.691 ± 0.105	0.823 ± 0.040	69.565	0.680	0.795
SVM	72.387 ± 4.543	0.696 ± 0.098	0.825 ± 0.027	72.174	0.694	0.818
Decision Trees	72.395 ± 4.307	0.624 ± 0.070	0.819 ± 0.032	71.304	0.646	0.811
Random Forest	72.689 ± 5.188	0.689 ± 0.118	0.825 ± 0.033	69.565	0.695	0.798
Logistic Regression	70.933 ± 3.874	0.698 ± 0.111	0.814 ± 0.028	72.174	0.704	0.814
Voting	73.546 ± 4.232	0.701 ± 0.115	0.831 ± 0.027	72.174	0.715	0.818
LSTM	74.135 ± 5.437	0.702 ± 0.115	0.828 ± 0.023	72.174	0.722	0.818

### Feature importance

**Fig. 12:**
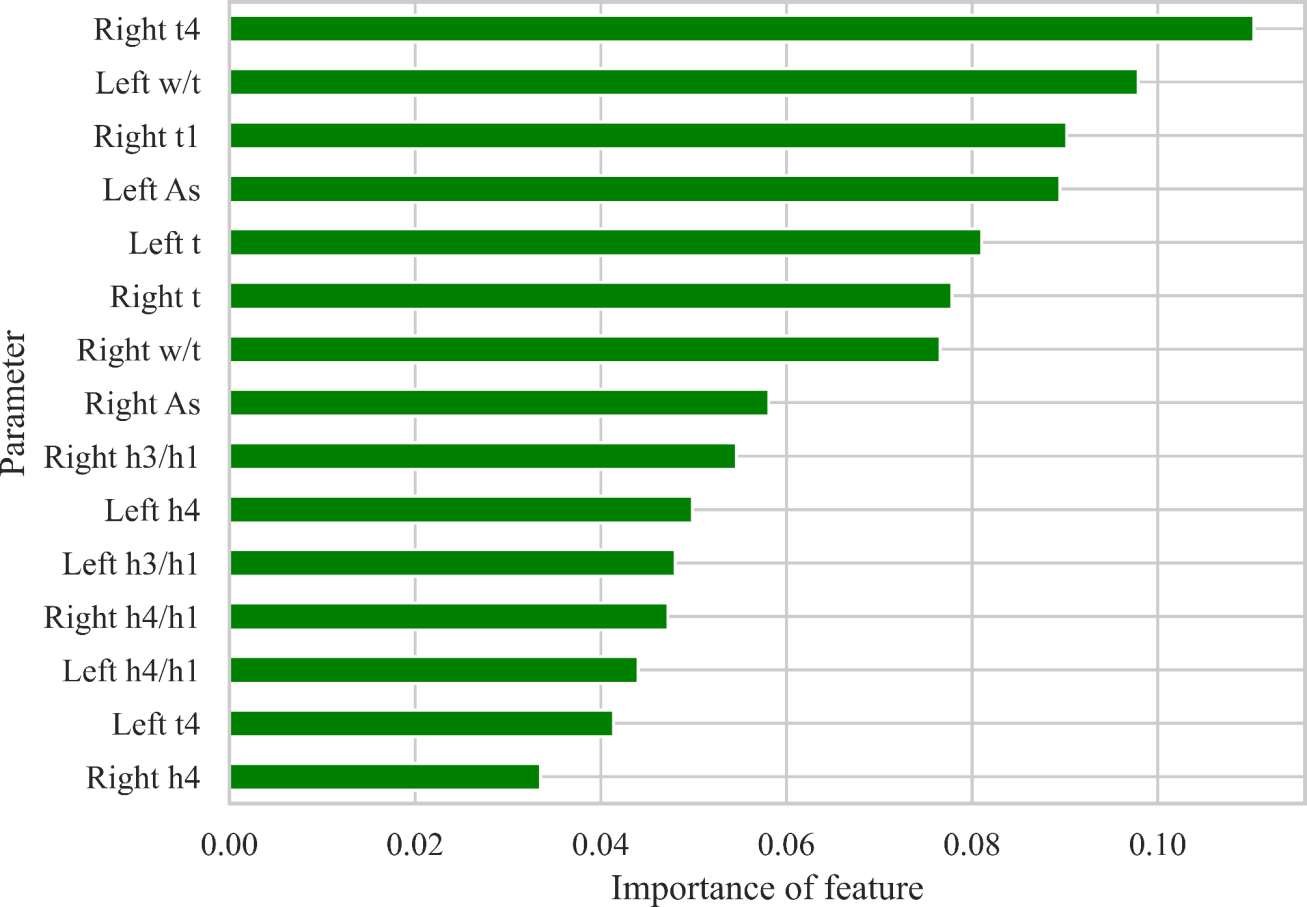
Feature importance of 15 features based on Random Forest

The features contributing most to the Random Forest model were reported (Fig. 12). Random forest with training accuracy 72.689 ± 5.188%, AUC 0.689 ± 0.118, and F1 score 0.825 ± 0.033. The top five features are right t4, left w/t, right t1, left As, and left t, these features contributed to 46.89% of importance over all features.

## Discussion

The PCOS group and the healthy group were age-matched (*P* > 0.05) but the BMI of the PCOS group was higher (*P* < 0.01) due to its metabolism disorders. The pulse wave parameters h3/h1, w/t, h4, t4, t, As, h4/h1 from both sides and right t1 were significantly difference between groups (*P* < 0.01). Compared to healthy individuals, PCOS patients experienced poorer vascular compliance, faster heart rate, and reduced left ventricular systolic function. Results of ML classification proved that pulse signal analysis could be used to predict PCOS patients, among the seven models, LSTM achieved the best testing performance.

Radial pulse wave is affected by the pulsation of the heart and conditions of arteries, tissues, and organs; thus, it could contain rich physiological and pathological information about individuals [37]. Time-domain analysis, which is one of the most widely used methods [9], was applied in this study to determine the correlation between PCOS and radial pulse wave parameters.

The metabolic disorder of PCOS causes vascular function changes. Studies reported that PCOS patients had evidence of an increased risk of hypertension [38], proven early atherosclerosis, and endothelial dysfunction, thus, increasing the risk of developing cardiovascular disease (CVD) compared to healthy women [39]. The relative risk for myocardial infarction is 7.4 for PCOS patients [40]. Radial pulse wave analysis can provide valuable information on the cardiovascular health of PCOS women.

From the results of this study, we found that the pulse wave parameters h3/h1 and w/t from both left and right sides were significantly higher in the PCOS group than in the healthy group, meanwhile, parameters right t1, and h4, t4, t, As, h4/h1 from both sides were significantly lower in PCOS group. Higher h3/h1 and w/t reflect poorer arterial elasticity or higher peripheral resistance in the PCOS group. Liu et al. found that h3/h1 was higher in the mild and severe occlusion group of coronary heart disease (CHD) patients compared to the non-occlusion group [41], which proved that high h3/h1 is related to poor vascular compliance, a risk factor of CVD.

Parameter t1 corresponds to the rapid ejection period, t4 reflects the systolic time of the left ventricle, and t is the time of a cardiac cycle of the left ventricle, therefore lower in t1, t4 and t indicates a shorter systolic phase and a faster heart rate of PCOS patient. As is the area of the systolic phase, lower As in the PCOS group suggests a decrease in cardiac output, which may result in insufficient peripheral tissue perfusion. Our result agreed with the finding that suggested PCOS women had lower left ventricular ejection fraction [42]. Parameters h4 and h4/h1 reflect the peripheral resistance of the artery, lower h4, and h4/h1 in the PCOS group probably because of decreasing in peripheral blood volume, as a result, the heartbeat increased compensatory. In brief, PCOS patients experienced poorer vascular compliance, faster heart rate, and reduced left ventricular systolic function compared to healthy individuals.

From the feature importance analysis of Random Forest, we noted that the top five features are right t4, left w/t, right t1, left As, and left t, these features contributed to 46.89% of importance over all 15 features for classification, the features are related to left ventricular function directly or indirectly. This result revealed that the left ventricle function is the prominent factor to distinguish PCOS patients from healthy individuals. The correlation between PCOS patients and left ventricular function is worth further exploration, previous research had also shown that PCOS women are associated with a higher left ventricular mass index and larger left atrial diameter [43]. For the t1 differed significantly only on the right wrist, we assumed that this is because the right wrist is more sensitive than the left wrist upon pulse wave detection, a study demonstrated that the predictive power of physical factors from the right wrist was higher than that of the left wrist [44].

During ML modelling, to reduce the data imbalanced effect, we applied a stratified k-fold cross-validation method. Then, the models were tuned by the grid search with cross-validation to get the best parameters. The cross-validations were repeated 10 times to ensure reliable results. Seven models were trained sequentially including KNN, SVM, Decision Trees, Random Forest, Logistic Regression, Voting, and LSTM. Rui Guo et al. found that SVM with Gaussian radial basis function is an effective tool for solving pattern recognition and function estimation problems and is suitable for pulse wave analysis [45]. On the other hand, Ding et al. found that Logistic Regression achieved the most satisfactory result among others in waveform classification [46], Logistic Regression classifier is consistent with the physiological process of the pulse wave. Su et al. proved that Random Forest could obtain higher accuracy in disease prediction due to its bootstrap aggregation and randomization of predictors, and it is less prone to overfitting [25]. To improve the model performance, we applied ensemble learning methods by using a Voting classifier, which made decision based on the highest average probability given to certain class from the first five models. LSTMs are suitable for capturing the temporal patterns present in the pulse wave parameter data studied [47, 48].

Our findings showed that these prediction models indicated similar performance in classifying the PCOS group and healthy group overall, but the Voting and LSTM did surpass the others. LSTM, a deep learning model known for its ability to capture temporal dependencies, demonstrated competitive performance across all metrics. It was the best-performing model on testing data, which achieved an accuracy of 72.174%, an AUC of 0.722, and an F1 score of 0.818. The Voting ensemble, composed of base models KNN, SVM, Decision Trees, Random Forest, and Logistic Regression, also performed remarkably well. It achieved an accuracy of 72.174%, an AUC of 0.715, and an F1 score of 0.818. Notably, both have the best performance across all training and testing metrics, showcasing their potential for effective modelling. The F1 scores from LSTM and Voting are considered at a good level, which means that the models can predict most of the PCOS cases and be accurate with them. However, the values of accuracy and AUC are only moderate and far from excellent compared to the other ML disease classifiers (featuring pulse parameters) as mentioned previously. The lower accuracy and AUC may be caused by the limitations including relatively small sample sizes (n = 459), and an imbalanced dataset (70% PCOS cases: 30% healthy cases).

In overall, the performance metrics on the testing data are generally slightly lower than those on the training data. This is expected as models tend to perform slightly worse on unseen data. None of the models seems to show significant overfitting, as the drop in performance from training to testing data is relatively small, and overfitting would occur only when a model performs well on training data but poorly on testing data.

Considering computational efficiency and simplicity, Voting might be the preferred option for model selection. It is important to note that LSTM, as a deep learning model, is a more computationally expensive model compared to Voting. LSTM training involves complex backpropagation through time steps, and hyperparameter tuning can contribute to its computational cost. On the other hand, Voting comprising simpler base models generally requires less computation.

The correlation between pulse diagnosis and PCOS was again clarified in this study. By comparing the different prediction models, the results could provide a reference for other clinical research. This study is subject to several limitations, comprising (1) even though the participants were instructed to remain calm and refrain from eating or drinking 30 min prior to the test, it is still possible for biases to be introduced due to factors such as patient tremors, respiration, mechanical vibrations from instruments, and power frequency interference; (2) although we debugged the equipment to a certain extent and tried our best to ensure a noise-free environment during data collection, the current level of science and technology still has certain limitations to fully distinguish noise from pulse signals; (3) a relatively small sample size, we used ensemble methods, cross-validation, and hyperparameter tuning to overcome the limitation caused by small sample size to a certain extent, and the results were verified that there was no overfitting. However, we will expand the sample size in the future to provide more reliable assessment results. In the follow-up study, we will increase sample sizes, balance the dataset, integrate pulse wave analysis with frequency domain parameters, and investigate the relationship between pulse conditions with different PCOS phenotypes. To increase the prediction ability in a way of TCM diagnostics, we suggest: (1) the diversifying of the pulse wave analysis methods; (2) the integration of the pulse data with tongue data and/or TCM symptoms.

In conclusion, there were significant differences in radial pulse waves between PCOS patients and healthy individuals. ML classification based on pulse wave analysis could identify most PCOS patients accurately with good F1 score and is valuable for early detection and monitoring of PCOS with acceptable overall accuracy. Voting classifier and LSTM with ensemble learning ability gave the best model performance among others. This radial pulse wave-based ML prediction method can stimulate the development of individualized PCOS risk assessment using mobile detection technology, with the advantages of being simple, convenient, non-invasive, and cost-effective. Nonetheless, this study gives physicians an intuitive understanding of the objective pulse diagnosis in TCM.

### Electronic supplementary material

Below is the link to the electronic supplementary material.

Supplementary Material 1

## Data Availability

The datasets generated and/or analysed during the current study are not publicly available due to ethical concern but are available from the corresponding author on reasonable request.

## Abbreviations

AUC: Area under the Receiver Operating Characteristic curve
BMI: Body mass index
CART: Classification and Regression Trees
CHD: Coronary heart disease
CVD: Cardiovascular disease
DT: Decision Trees
FN: False negatives
FP: False positives
KNN: K-Nearest Neighbors
LR: Logistic Regression
LSTM: Long Sort Term Memory networks
ML: Machine learning
PCOM: Polycystic ovarian morphology
PCOS: Polycystic ovary syndrome
RBF: Radial basis function
RF: Random Forest
RNN: Recurrent neural network
ROC: Receiver Operating Characteristic
SHUTCM: Shanghai University of Traditional Chinese Medicine
SV: Soft Voting
SVM: Support Vector Machine
TCM: Traditional Chinese Medicine
TN: True negatives
TP: True positives
TRIPOD: Transparent Reporting of a Multivariable Prediction Model for Individual Prognosis or Diagnosis
