# Supplementary material for: Machine learning classification of polycystic ovary syndrome based on radial pulse wave analysis
Source: BMC Complement Med Ther. 2023 Nov 13;23:409. doi: 10.1186/s12906-023-04249-5 (PMC10644435; doi:10.1186/s12906-023-04249-5)
Supplement: Supplementary file 1 — Supplementary Material 1 [file 12906_2023_4249_MOESM1_ESM.docx]

**Supplementary Material**

Table S1. Machine learning classifier and its hyperparameter setting (coding with Python Scikit-Learn version 1.2.2)

| **Clasifier** | **Hyperparamters/Coefficients** |
| --- | --- |
| K Nearest Neighbors:  KNeighborsClassifier() | {'algorithm': 'auto', 'leaf_size': 30, 'metric': 'minkowski', 'metric_params': None, 'n_jobs': None, 'n_neighbors': 22, 'p': 2, 'weights': 'uniform'} |
| Support Vector Machine:  SVC() | {'C': 1.0, 'break_ties': False, 'cache_size': 200, 'class_weight': None, 'coef0': 0.0, 'decision_function_shape': 'ovr', 'degree': 3, 'gamma': 0.05, 'kernel': 'rbf', 'max_iter': -1, 'probability': True, 'random_state': None, 'shrinking': True, 'tol': 0.001, 'verbose': False} |
| Decision trees:  DecisionTreeClassifier() | {'ccp_alpha': 0.0, 'class_weight': None, 'criterion': 'gini', 'max_depth': 2, 'max_features': None, 'max_leaf_nodes': None, 'min_impurity_decrease': 0.0, 'min_samples_leaf': 1, 'min_samples_split': 2, 'min_weight_fraction_leaf': 0.0, 'random_state': 1, 'splitter': 'best'} |
| Random forest:  RandomForestClassifier() | {'bootstrap': True, 'ccp_alpha': 0.0, 'class_weight': None, 'criterion': 'gini', 'max_depth': 4, 'max_features': 'sqrt', 'max_leaf_nodes': None, 'max_samples': None, 'min_impurity_decrease': 0.0, 'min_samples_leaf': 1, 'min_samples_split': 2, 'min_weight_fraction_leaf': 0.0, 'n_estimators': 300, 'n_jobs': None, 'oob_score': False, 'random_state': 1, 'verbose': 0, 'warm_start': False} |
| Logistic regression:  LogisticRegression() | {'C': 1.0, 'class_weight': None, 'dual': False, 'fit_intercept': True, 'intercept_scaling': 1, 'l1_ratio': None, 'max_iter': 100, 'multi_class': 'auto', 'n_jobs': None, 'penalty': 'l2', 'random_state': 1, 'solver': 'lbfgs', 'tol': 0.0001, 'verbose': 0, 'warm_start': False}  Coefficients:  Left h4: 0.0666, Left t4: 0.1803, Left t: -0.1104, Left as: -0.2726, Left h3/h1: -0.0159, Left h4/h1: -0.067, Left w/t: 0.3601, Right h4: 0.0371, Right t1: -0.9188, Right t4: -0.2643, Right t: -0.1714, Right as: -0.1384, Right h3/h1: -0.2683, Right h4/h1: -0.046, Right w/t: 0.4727 |
| Voting:  VotingClassifier() | {'estimators': [('K Nearest Neighbours', KNeighborsClassifier(n_neighbors=22)), ('SVM', SVC(gamma=0.05, probability=True)), ('Decision Tree', DecisionTreeClassifier(max_depth=2, random_state=1)), ('Random Forest', RandomForestClassifier(max_depth=4, n_estimators=300, random_state=1)), ('Logistic Regression', LogisticRegression(random_state=1))], 'flatten_transform': True, 'n_jobs': None, 'verbose': False, 'voting': 'soft', 'weights': None, 'K Nearest Neighbours': KNeighborsClassifier(n_neighbors=22), 'SVM': SVC(gamma=0.05, probability=True), 'Decision Tree': DecisionTreeClassifier(max_depth=2, random_state=1), 'Random Forest': RandomForestClassifier(max_depth=4, n_estimators=300, random_state=1), 'Logistic Regression': LogisticRegression(random_state=1), 'K Nearest Neighbours__algorithm': 'auto', 'K Nearest Neighbours__leaf_size': 30, 'K Nearest Neighbours__metric': 'minkowski', 'K Nearest Neighbours__metric_params': None, 'K Nearest Neighbours__n_jobs': None, 'K Nearest Neighbours__n_neighbors': 22, 'K Nearest Neighbours__p': 2, 'K Nearest Neighbours__weights': 'uniform', 'SVM__C': 1.0, 'SVM__break_ties': False, 'SVM__cache_size': 200, 'SVM__class_weight': None, 'SVM__coef0': 0.0, 'SVM__decision_function_shape': 'ovr', 'SVM__degree': 3, 'SVM__gamma': 0.05, 'SVM__kernel': 'rbf', 'SVM__max_iter': -1, 'SVM__probability': True, 'SVM__random_state': None, 'SVM__shrinking': True, 'SVM__tol': 0.001, 'SVM__verbose': False, 'Decision Tree__ccp_alpha': 0.0, 'Decision Tree__class_weight': None, 'Decision Tree__criterion': 'gini', 'Decision Tree__max_depth': 2, 'Decision Tree__max_features': None, 'Decision Tree__max_leaf_nodes': None, 'Decision Tree__min_impurity_decrease': 0.0, 'Decision Tree__min_samples_leaf': 1, 'Decision Tree__min_samples_split': 2, 'Decision Tree__min_weight_fraction_leaf': 0.0, 'Decision Tree__random_state': 1, 'Decision Tree__splitter': 'best', 'Random Forest__bootstrap': True, 'Random Forest__ccp_alpha': 0.0, 'Random Forest__class_weight': None, 'Random Forest__criterion': 'gini', 'Random Forest__max_depth': 4, 'Random Forest__max_features': 'sqrt', 'Random Forest__max_leaf_nodes': None, 'Random Forest__max_samples': None, 'Random Forest__min_impurity_decrease': 0.0, 'Random Forest__min_samples_leaf': 1, 'Random Forest__min_samples_split': 2, 'Random Forest__min_weight_fraction_leaf': 0.0, 'Random Forest__n_estimators': 300, 'Random Forest__n_jobs': None, 'Random Forest__oob_score': False, 'Random Forest__random_state': 1, 'Random Forest__verbose': 0, 'Random Forest__warm_start': False, 'Logistic Regression__C': 1.0, 'Logistic Regression__class_weight': None, 'Logistic Regression__dual': False, 'Logistic Regression__fit_intercept': True, 'Logistic Regression__intercept_scaling': 1, 'Logistic Regression__l1_ratio': None, 'Logistic Regression__max_iter': 100, 'Logistic Regression__multi_class': 'auto', 'Logistic Regression__n_jobs': None, 'Logistic Regression__penalty': 'l2', 'Logistic Regression__random_state': 1, 'Logistic Regression__solver': 'lbfgs', 'Logistic Regression__tol': 0.0001, 'Logistic Regression__verbose': 0, 'Logistic Regression__warm_start': False} |

Table S2. Long Sort Term Memory networks (LSTM) model architecture and hyperparameters

| **Model** | **Architecture/Hyperparameters** |
| --- | --- |
| LSTM architecture | model = Sequential()  model.add(LSTM(32, input_shape = (1, 15), activation = 'relu', return_sequences=True))  model.add(Dropout(0.2))  model.add(LSTM(16))  model.add(Dropout(0.2))  model.add(Dense(16, activation='relu'))  model.add(Dropout(0.2))  model.add(Dense(1, activation='sigmoid'))  model.compile(optimizer = Adam(learning_rate=0.001)  , loss = 'binary_crossentropy', metrics = ['accuracy']) |
| KerasClassifier() | epochs = 50, batch_size = 128, random_state=70, shuffle=None |
